# Korean Medication Algorithm Project for Bipolar Disorder 2022: Treatment Strategy According to Safety and Tolerability

**DOI:** 10.1192/j.eurpsy.2024.1225

**Published:** 2024-08-27

**Authors:** S. Y. Park, W.-M. Bahk, Y. S. Woo, D.-I. Jon, M.-D. Kim, I. Sohn

**Affiliations:** ^1^Department of Psychiatry, Keyo Hospital, Uiwang; ^2^Department of Psychiatry, Yeouido St. Mary’s Hospital, College of Medicine, The Catholic University of Korea, Seoul; ^3^Department of Psychiatry, Hallym University Sacred Heart Hospital, Hallym University College of Medicine, Anyang; ^4^Department of Psychiatry, Jeju National University Hospital, Jeju, Korea, Republic Of

## Abstract

**Introduction:**

Accordingly, the Korean Medication Algorithm Project for Bipolar Disorder (KMAP-BP) working committee, composed of domestic experts, developed Korea’s first KMAP-BP in 2002 and later in 2006, 2010, and 2010. A revised version of KMAP-BP was announced every four years four times in 2014 and 2018.6-10). The treatment strategy considering the safety and tolerability of KMAP-BP 2022 was developed by collecting opinions from domestic bipolar disorder experts.

**Objectives:**

Safety and tolerability of drugs are very important factors in the treatment of bipolar disorder. An expert opinion survey was conducted on treatment strategies in various special clinical situations, such as significant weight gain, characteristic drug side effects, low drug adherence, pregnant and reproductive women, and genetic counseling.

**Methods:**

A written survey about treatment strategies related to safety and tolerability was prepared and focused on significant weight gain, characteristic drug side effects, low drug adherence, pregnant and reproductive women, and genetic counseling. Ninety-three experts of the review committee completed the survey.

**Results:**

In the case of weight gain occurring during drug treatment, it was preferred to replace it with a drug that caused less weight gain, such as lamotrigine, aripiprazole, or ziprasidone. If there was a significant weight gain due to the treatment drug, it was preferred to intervene as soon as possible. In the case of hyperprolactinemia, it was selected to change the medication and discontinue it for benign rash caused by lamotrigine. In improving drug adherence, the preference for long-acting injections increased. Antipsychotics can be used with great caution in pregnant or reproductive women.

**Conclusions:**

Treatment strategies in various clinical situations related to safety and tolerability in drug treatment for bipolar disorder were described. It is hoped that it will be useful in practical clinical situations.

**Disclosure of Interest:**

None Declared

